# A Continuous Assay Set to Screen and Characterize Novel Protein N-Acetyltransferases Unveils Rice General Control Non-repressible 5-Related N-Acetyltransferase2 Activity

**DOI:** 10.3389/fpls.2022.832144

**Published:** 2022-02-22

**Authors:** Thomas Asensio, Cyril Dian, Jean-Baptiste Boyer, Frédéric Rivière, Thierry Meinnel, Carmela Giglione

**Affiliations:** Institute for Integrative Biology of the Cell (I2BC), Université Paris-Saclay, CEA, CNRS, Gif-sur-Yvette, France

**Keywords:** N-acetyltransferase, acetylation, modifications, GNAT, rice, yeast, NatA

## Abstract

Protein N-acetyltransferases (NATs) belong to the general control non-repressible 5 (Gcn5)-related N-acetyltransferases (GNATs) superfamily. GNATs catalyze the transfer of acetyl from acetyl-CoA to the reactive amine moiety of a wide range of acceptors. NAT sequences are difficult to distinguish from other members of the GNAT superfamily and there are many uncharacterized GNATs. To facilitate the discovery and characterization of new GNATs, we have developed a new continuous, non-radioactive assay. This assay is virtually independent of the substrate and can be used to get substrate specificity hints. We validated first the assay with the well-characterized *Schizosaccharomyces pombe* NatA (SpNatA). The SpNatA kinetic parameters were determined with various peptides confirming the robustness of the new assay. We reveal that the longer the peptide substrate the more efficient the enzyme. As a proof of concept of the relevance of the new assay, we characterized a NAA90 member from rice (*Oryza sativa*), OsGNAT2. We took advantage of an *in vivo* medium-scale characterization of OsGNAT2 specificity to identify and then validate *in vitro* several specific peptide substrates. With this assay, we reveal long-range synergic effects of basic residues on OsGNAT2 activity. Overall, this new, high-throughput assay allows better understanding of the substrate specificity and activity of any GNAT.

## Introduction

Protein modifications involve a huge number (>400) of different chemical groups and acceptor places where the modifiers may act^1^ (Khoury et al., 2011). Acetylation on reactive amino groups is among the most frequent protein modifications together with phosphorylation of side chain hydroxyl groups (Aebersold and Mann, 2016). Protein acetylations are ensured by protein N-acetyltransferases. Acetylations can occur either at the protein N-termini (NTA) or on internal lysine side chains (KA) of proteins, peptides, or single amino acids. It was long surmised that NTA and KA were supported by distinct families of enzymes (NATs and KATs, respectively). Interestingly, recent data have revealed that a number of N-acetyltransferases display both specificities (KNATs; Aksnes et al., 2019; Bienvenut et al., 2020; Linster et al., 2020; Giglione and Meinnel, 2021). NATs and KNATs all belong to the superfamily of general control non-repressible 5 (GCN5)-related N-acetyltransferases (GNATs), whereas only one group among the three types of KATs corresponds to GNATs; the other KATs reported so far belong to the MYST, and p300/CBP families (Berndsen and Denu, 2008; Drazic et al., 2016). GNATs share very low overall sequence homology (3%–23%) but display conserved secondary and 3D structures (Dyda et al., 2000; Vetting et al., 2005; Salah Ud-Din et al., 2016). GNATs include proteins all featuring acyl transfer from a pantothenate-containing derivative donor, usually AcetylCoA (Ac-CoA). Although the GNAT domain has diverged during evolution, a general amino acid profile often identifies proteins belonging to the GNAT superfamily. Protein acetyltransferases of the GNAT family, including NATs, KATs, and KNATs, are difficult to distinguish among them and from the other members of the GNAT superfamily that encompass a number of small metabolites as acetyl receptors, including antibiotics, such as chloramphenicol, amino acids, sugars etc. (Vetting et al., 2005). Screening for GNATs in the genome database reveals that there are many uncharacterized GNATs (Rathore et al., 2016; Krtenic et al., 2020) but identifying the associated specificity/target is still most challenging. As an illustration, the characterization of rice serotonin acetyltransferase required the screening of as many as 31 open reading frames in *Escherichia coli* (Kang et al., 2013).

Various types of NATs (Deng and Marmorstein, 2021) including eight in Eukaryotes and four in Prokaryotes have been progressively defined, each displaying specific features including the involvement or not of auxiliary subunits for three of them and different substrate specificity on protein targets (Aksnes et al., 2016, 2019). Each NAT exhibits at least one catalytic core that may be associated or not with one and up to four auxiliary subunits that do influence both selectivity and efficiency of the reaction. For instance, the NatA complex from fungi is made up of the NAA10 small catalytic core (26 kDa), which displays the GNAT fold and of the NAA15 auxiliary large subunit (95 kDa; Deng and Marmorstein, 2021). Auxiliary subunits do not display the GNAT fold. Nevertheless, only NatA heterodimers recapitulate *in vitro* the selectivity observed *in vivo* in yeast for proteins starting with small N-terminal residues mostly, including Ala, Ser, or Thr—and Cys, Gly, and Val to a lesser extent—and features relevant catalytic parameters (Aksnes et al., 2016). Systematic screening approaches for identifying new NATs have progressively defined further catalysts.

We recently defined a new family—NatG—uniquely localized in plastids, with two distinct catalyst subtypes, NAA70 and NAA90 each featuring KNAT activity (Giglione and Meinnel, 2021). So far, NatG members have been only characterized in dicots but they were also predicted to occur in all plants including monocots and all non-seed plants. A NatG homolog could be identified in the unicellular alga *Chlamydomonas reinhardtii* (Westrich et al., 2020). NatG members display very relaxed specificity, featuring both NAT and KAT activity (Bienvenut et al., 2020). In the course of this discovery, the Global Analysis Profiling (GAP) Assay (Dinh et al., 2015) was applied to each of the eight NatG candidates to map their overall NTA activity and substrate specificity (Bienvenut et al., 2020). A modified version of the GAP assay was used to define the KA activity and substrate specificity of the same NatG members. In a separate approach, the relative specific activities *in vitro* of the NTA or KA activities could be measured for one member of each of NAA70 (AtGNAT10) and NAA90 (AtGNAT2) using partially purified Maltose Binding Protein (MBP) fusions. To do so, a HPLC-based assay with specific peptides was used. Such peptide substrates display an N-terminal octapeptide featuring a fluorophore (2,4-dinitrophenyl) linked on the ε-NH_2_ of a C-terminal Lys residue and an increased solubility ensured by a C-terminal linker featuring aminohexanoic acid extended with a tail of D-Arginines (Seidel et al., 2016; Bienvenut et al., 2020). Acetylated products display increased elution times on reverse phase chromatography. HPLC based assay was also used to monitor NTA with human NAA30 (Evjenth et al., 2009, 2013) and the *Arabidopsis thaliana* NatA complex (Linster et al., 2015). In addition to the above assays, two additional assays are commonly used to assess the activity of N-acetyltransferases. The most common one involves radiolabeled ^14^C- or ^3^H-Ac-CoA and peptides displaying a strong basic character allowing their retention on anionic filters (Gottlieb and Marmorstein, 2019; Linster et al., 2020). Though some substrates may naturally contain strongly basic features like the histone H4-derived substrate of NatD (Magin et al., 2015), a polybasic C-terminal track of 12 residues is usually added to a heptapeptide, which sequence can be modified according to the expected specificity of each NAT. Another assay consists in the use of 5,5′-dithiobis-(2-nitrobenzoic acid) (DTNB). DTNB reacts with the thiol moiety of the CoA released from Ac-CoA and produces 2-nitro-5-thiobenzoate (TNB). TNB absorbance is followed over time. This assay has the unique advantage that it allows continuous measurements with almost any polypeptide sequence. The major drawback is that DTNB prevents any thiol-containing compound—like Cys or 2-mercaptoethanol—and strong reducers to occur in the reaction mixture that would produce otherwise TNB, blurring NTA kinetics as a result. Use of this assay is therefore precluded for any enzyme which preparation requires addition of a reducing agent to preserve its activity. As DTNB detection is also not very sensitive below 10 μM concentration ranges, the fluorescent probe ThioGlo4 that makes specific adducts with the CoA products was introduced successfully to assay NatD (Ho et al., 2021).

Here, we report the development of a continuous high throughput assay, which is workable with Ac-CoA as acetyl donor and any acceptor substrate including amino acids or short peptides, independently of their amino acid sequence. In this assay, GNAT activity is coupled with that of pyruvate dehydrogenase (PDH), which uses the released CoA and produces NADH. We validated the assay with *Schizosaccharomyces pombe* NatA (SpNatA) and revealed that substrate length and polybasic tail strongly influence the SpNatA kinetics. As a proof of concept of the versatility of the assay, we characterized the NTA kinetic properties of a putative plastid GNAT from the monocotyledonous crop *Oryza sativa* (rice), as land-plants appear to display a remarkably rich panel of GNATs. Our data show that OsGNAT2 displays similar specific activity to the recently reported homolog counterpart from the dicotyledonous model plant *A. thaliana*, demonstrating its applicability for any putative GNAT independently from the species. As with SpNatA, we also observed that remote polybasic tracks contribute to improve OsGNAT2 kinetics.

## Materials and Methods

### Chemicals

All peptides (see all sequences in Supplementary Table 1) were purchased at 95% purity (Genscript, Leiden, Netherlands). NAD was purchased from Roche (Basel, Switzerland). All other chemicals were purchased from Sigma-Aldrich (St. Quentin, France).

### Cloning and Purification of SpNatA

The full length (FL) sequence of SpNaa10 (177 residues) from pETDuet-SpNaa10(FL) was introduced between the *Nde*I and *Kpn*I restriction sites of pETDuet-SpNaa15(FL)-SpNaa10(1–156; Liszczak et al., 2013) to replace the 1–156 truncated open reading frame with the FL and produce pETDuet-SpNaa15(FL)-SpNaa10(FL). Purification of SpNatA was as described in Gottlieb and Marmorstein (2019) with the exception of the last step of size exclusion chromatography, which was skipped as proven unnecessary for the sake of enzyme assays. Purity was assessed >99%.

### Cloning and Purification of Fusion OsGNAT2-MBP

The DNA fragment encoding amino acid residues 89–254 of GNAT2 from *O. sativa* (UniProt ID Q5KQI6) was inserted between the *Nco*I/*Bam*HI restriction sites of the pETM41 vector featuring a dual N-terminal hexahistidine-Mannose binding protein (MBP) tag followed by a Tobacco Etch Virus (TEV) cleavage site. This plasmid (pETM41-OsGNAT2) was propagated in *E. coli* BL21-pLysS Rosetta2 cells (Novagen). After 0.25 mM IPTG induction at OD_600_ = 0.6, cells were gown overnight for 20 h at 20°C in 2YT medium. Cells were lysed by sonication at 4°C in 20 mM Tris–HCl (pH 8.0), 0.5 M NaCl, and 5 mM 2-mercaptoethanol supplemented with 10 mM imidazole (Buffer A). The lysate was loaded directly onto an immobilized nickel ion affinity chromatography (HisTrap Crude FF, 5 ml, GE Healthcare) and protein eluted by a linear gradient of 10–250 mM imidazole in buffer A (2 ml/min, 100 ml). The pool of purified protein was next dialyzed 24 h against the storage buffer containing 20 mM Tris-HCl (pH 8.0); 0.2 M NaCl, 1 mM 1,4-Dithiothreitol (DTT), and 55% glycerol (Buffer B). The 6xHis-MBP-OsGNAT2 protein samples were stored at −20°C.

For preparation of OsGNAT2 devoid of MBP, the sample obtained after the aforementioned nickel affinity purification was further dialyzed overnight against a buffer containing 20 mM Tris–HCl (pH 8.0), 0.2 M NaCl, and 5 mM 2-mercaptoethanol supplemented with 10 mM imidazole (Buffer C) in the presence of 1 mg per 15 mg purified protein of a TEV protease version displaying a C-terminal polyhistidine-tag (To et al., 2015). The sample was next applied onto a nickel affinity column (HisTrap Crude FF, 5 ml, GE Healthcare), and the column was washed with 25 ml buffer C. The flow-through—corresponding to OsGNAT2 devoid of both the 6xHis-MBP tag and TEV-6xHis—was diluted four times in buffer D containing 20 mM Tris–HCl (pH 8.0), 1 mM DTT. The OsGNAT2 sample was loaded on an anion exchange column (HiTrap Q FF 5 ml), and the protein was eluted using a linear gradient of 0–1 M NaCl in buffer A (2 ml/min, 75 ml). Pulled fractions of the OsGNAT2 protein were then dialyzed against buffer B and stored at −20°C.

### Global Acetylation Profiling

Plasmids used for the experiment were pETM41-OsGNAT2 (This work) for OsGNAT2 induction and pETM41-AtNAA15 for control induction as NAA15 does not have any acetylase activity (Linster et al., 2015). Cultures were grown in 30 ml LB media at 37°C until OD_600_ reaches a value of 0.2. Next, induction was promoted by adding of 0.25 mM IPTG. Cultures were grown for another two generations or so in the exponential phase (OD_600_ = 0.9–1). Cells were harvested, frozen in liquid nitrogen, and stored at −80°C. The sample was solubilized in 300 μl of a buffer containing 50 mM HEPES/NaOH pH 7.2; 1.5 mM MgCl_2_; 1 mM EGTA; 10% glycerol; 1% Triton X-100; 0.15 M NaCl; 2 mM phenylmethylsulfonyl fluoride (PMSF); and one tablet of protease inhibitor cocktail (EDTA+; Merck) and was lysed by ultra-sonication. All further steps are reported in details in Bienvenut et al. (2020) and were performed without any deviation. The SILProNAQ pipeline for acetylation yield measurements is detailed in Bienvenut et al. (2017a,b).

### PDH-Coupled Assay of GNAT

GNATs activity was assayed at 30°C in a coupled assay with PDH (see reactions 1 and 2 in the section “Results and Discussion”). The reaction mixture contained 50 mM Tris–HCl (pH 8.0), 100 mM NaCl, 1 mM MgCl_2_, 1 mM EGTA, 1 mM DTT, 0.2 mM thiamine pyrophosphate, 2 mM pyruvate, 2.5 mM NAD^+^, 0.150 units/ml of porcine heart PDH, 10–500 μM Ac-CoA (>93% pure, Sigma, A2056), and 10–5,000 μM peptides (Genscript). The reaction mixture was pre-incubated for 5 min at 30°C before starting the reaction by adding 10–500 μM Ac-CoA. A final volume of 200 μl was used in 96-well black plates (Grenier Bio One and Dominique Dutscher, Brumath, France; optical path for 0.2 ml is 0.44 cm). A value of 6,300 M^−1^.s^−1^ was used as the molecular extinction coefficient of NADH at 340 nm. An Infinite M Nano+ plate reader (Tecan, Lyon, France) was set at 340 nm to monitor the absorbance over time at 30°C.

Acetylation kinetics was monitored continuously for 5–120 min, and the data were fitted as to obtain the initial velocities associated to each peptide concentration. Curve fits to obtain kinetic parameters were achieved by non-linear regression with GraphPad Prism 9.1 (GraphPad Software, La Jolla, CA, United States). Parameters with SEs were computed for all parameters using the complete dataset including replicates. Both *k_cat_* and *K_m_* kinetic values were obtained by fitting to the Michaelis–Menten equation. When *V*_max_ could not be reached, the *k_cat_*/*K_m_* value was obtained with simple linear regression fit with Graphpad Prism 9.1.

### DTNB Coupled Assay

The overall incubation conditions were identical to those of the PDH-coupled assay (see before) but the coupling reagents (i.e., MgCl_2_, Pyruvate, NAD^+^, thiamine pyrophosphate, and PDH) and DTT were omitted. To ensure DTT would not challenge the reaction of DTNB with CoA, SpNatA was diluted strongly (>5,000-fold) from the 1 mM-DTT containing stock solution in reaction buffer without DTT leading to submicromolar concentration that eventually only poorly contributed to the signal. DTNB from a stock solution at 10 mM in ethanol was added at a final concentration of 0.1 mM in the incubation buffer. This corresponded to the best assay conditions compared to final DTNB concentrations of 1 mM obtained from 10 mM stock solutions solubilized either in ethanol or freshly prepared in potassium phosphate buffer solution, pH 7.2 containing 0.1 mM EDTA (Supplementary Figure 1). Absorbance was monitored continuously over time at 412 nm. A value of 13,600 M^−1^.s^−1^ was used as the molecular extinction coefficient of the thiophenolate (TNP) anion (Silverstein, 1975).

### MALDI-Tof Spectrometry

Matrix Assisted Laser Desorption Ionization Time-Of-Flight (MALDI-Tof) spectrometry was performed as described in Dian et al. (2020) as follows. About 100 μl of a mixture containing 100 mM Tris–HCl (pH 8.0), 200 mM NaCl, 1 mM EGTA, 1 mM DTT, 100 μM Ac-CoA, 5 μM OsGNAT2, and 0.5 mM of synthetic peptide TQTFIPGKDA (Genscript, Piscataway, NJ, United States) were incubated at 30°C for 60 min. Around 10 μl samples withdrawn before and after incubation were diluted in 90 μl of water/acetonitrile solution. The samples were then diluted five times in the matrix solution made of 5 mg/ml of α-cyano-4-hydroxycinnamic acid solubilized in water/formic acid/acetonitrile (50/50/0.1%). About 1 μl of each dilution was spotted on a metal target and dried. MS and MS/MS spectra of each sample were acquired with an AB SCIEX 5800 MALDI-Tof-Tof instrument in positive ion mode. Survey scans were performed using delayed extraction (390 ns) in reflector mode for a total of 15,000 shots. MS/MS scans were operated with collision energy of 1 kV. Peptide and fragment mass tolerances were set at 10 ppm and 0.8 Da, respectively. Mass spectra were analyzed with PeakView® 2.2 software (AB Sciex, Macclesfield, United Kingdom). The default threshold in MS/MS peak labelling and finding was 5%.

## Results and Discussion

### Setting the Guidelines for a GNAT-Coupled Assay With Pyruvate Dehydrogenase

N-acetyltransferases transfer the acetyl moiety from Ac-CoA to the N-terminal ammonium of peptide substrates. They release coenzyme A (CoA), which can be used by decarboxylating enzymes such as those of the Krebs cycle in the presence of NAD^+^ to produce NADH. NADH is often used in spectrometric assay as a reporter because of its ability—unlike NAD^+^—to strongly absorb light at 340 nm and fluoresce at 465 nm when excited at 340 nm. PDH or α-ketoglutarate dehydrogenase were already used successfully as coupling enzymes using pyruvate or α-ketoglutarate, respectively, to produce NADH. Examples involve histone, palmitoyl, or myristoyl acyl transferases (Boisson and Meinnel, 2003; Berndsen and Denu, 2005; Traverso et al., 2013; Hamel et al., 2014). When using Ac-CoA as the acyl donor of the primary reaction, PDH regenerates Ac-CoA. This offers the advantage over another dehydrogenase of keeping the second substrate (Ac-CoA) constant during the reaction time, improving initial velocity duration and avoiding any aberration of the associated kinetics caused, for example, by product inhibition. We chose therefore PDH as a valuable coupling enzyme to start with. GNAT activity (equation 1) was assayed by continuously monitoring the formation of NADH in a coupled assay using PDH activity (equation 2) as follows (where *R* is any chemical compound):


(1) $$\begin{array}{l} {\mathrm{RNH}}_{3}{}^{+}+\mathrm{Acetyl-CoA}+H_{2}O\to \\ \mathrm{Acetyl-NH-R}+\mathrm{CoA-SH}+H_{3}O^{+} \end{array}$$

(2) $$\begin{array}{l} \mathrm{Pyruvate}+\mathrm{CoA-SH}+{\mathrm{NAD}}^{+}\to \\ \mathrm{Acetyl-CoA}+{\mathrm{CO}}_{2}+\mathrm{NADH}+H_{3}O^{+} \end{array}$$

In order to set the conditions of the assay, we chose as model NAT SpNatA, an enzyme extensively studied in the past using the discontinuous radioactive test (Gottlieb and Marmorstein, 2019). In the radioactive assay, the model peptide is composed of a fusion of the N-terminal heptapeptide from the yeast threonyl-tRNA synthetase (Ths1p), which was previously shown to be an excellent substrate *in vivo* of yeast NatA (Polevoda and Sherman, 2003; Liszczak et al., 2013). This peptide was fused to a C-terminal 12-mer tag derived from AdrenoCorticoTropic Hormone (ACTH) featuring reinforced strong basic character—including three Lys to Arg substitutions—aimed at tightly retaining the peptide on anionic beads including phosphocellulose filter papers such as P81 or LSA-50 (Arnesen et al., 2009; Appelmans et al., 2021). This allows to separate the labeled acetyl group attached on the peptide product from the bulk of radiolabeled Ac-CoA, which is negatively charged and does not bind the filter. This19-amino acid peptide SASEAGV*RWGRPVGRRRRP* defines the canonical NatA substrate (reference peptide) that was used throughout this study.

In order to set a relevant, robust coupled enzymatic assay, the initial mandatory step was to determine all conditions allowing PDH not to be rate limiting; under such conditions the NADH readout over time only relies on the first reaction. We used a PDH concentration slightly above the value previously validated as facilitating kinetic data recovery (Traverso et al., 2013). Next, we challenged the SpNatA activity by progressive increase of its concentration in the presence of the reference peptide substrate (SASEAGV*RWGRPVGRRRRP*) and Ac-CoA at equimolar concentrations (0.5 mM). At SpNatA concentrations beyond 0.1 μM, the reaction rates were no longer proportional and resulted in a loss of apparent velocity rate indicating that PDH was limiting (Supplementary Figure 1A). The SpNatA rate measured in the linear part of the curve was in perfect agreement with the data previously published under the same conditions but obtained with the radioactive assay (2 vs. 1.8 s^−1^; Liszczak et al., 2013). With this information, we concluded that relevant rate measurements could be obtained provided the rate was lower than 0.2 μM.s^−1^. This rate limit was applied to all further experiments. Any rate reaction exceeding this value had to be performed again with a lower concentration of enzyme to slow down the reaction and make it compatible with non-limiting PDH rate.

**Figure 1:**
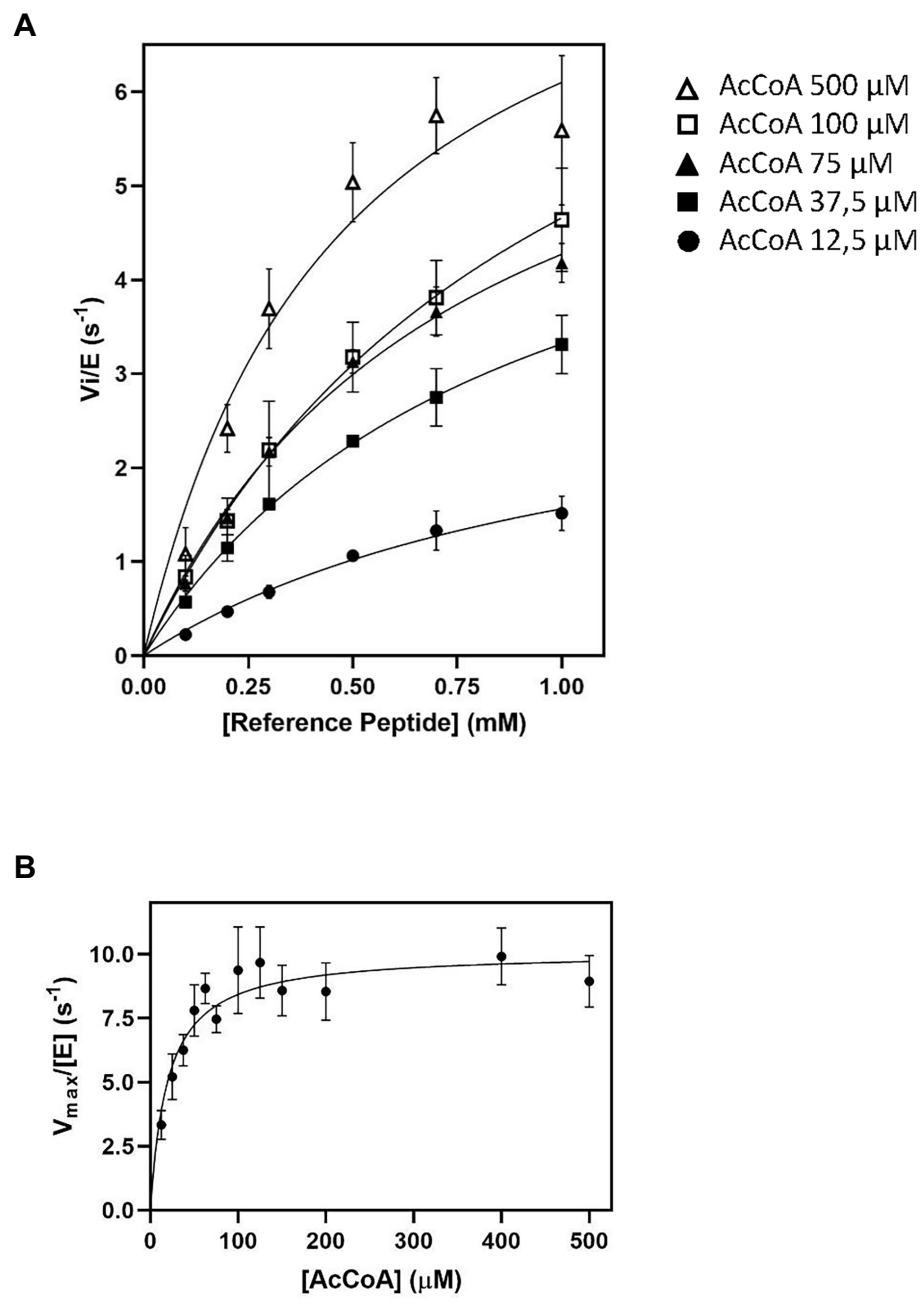
Impact of AcetylCoA (Ac-CoA) concentration on *Schizosaccharomyces pombe* NatA (SpNatA)-catalyzed acetylation as assessed with the pyruvate dehydrogenase (PDH)-coupled assay. The reference peptide (SASEAGVRWGRPVGRRRRP) was used (0.1–1 mM concentration range). Acetylation of was achieved in the presence of 0.01 μM SpNatA. The PDH-coupled assay was used for all measurements, and the data are the average of three independent experiments. Data were fitted to the Michelis–Menten equation, and the resulting curve is displayed as a continuous line. **(A)** Measurements performed in the presence of Ac-CoA concentrations of 12.5, 37.5, 75, 100, and 500 μM. **(B)** Kinetic curve of Michaelis–Menten equation fit of SpNatA catalysis with Ac-CoA. Each value originates from experiments at a given Ac-CoA concentration (see panels **A** for instance) where the maximum velocity rate was determined at varying reference peptide concentrations.

We next calculated the Michaelis–Menten kinetic parameters of the reference peptide using an array of concentrations ranging from 0.1 to 1 mM. We could calculate a *k_cat_* of 9 ± 1 s^−1^ and a *K_m_* of 0.47 ± 0.12 mM (Table 1; Figure 1A). These data, albeit close to the previously reported values of 3 s^−1^ and 0.34 mM, respectively, with the same peptide, feature a significantly higher *k_cat_* value and a similar *K_m_*. Higher *k_cat_* values obtained with a coupled assay as compared to a radioactive assay were already observed with other acetyltransferases (see Table 1 in Kim et al., 2000). One most likely explanation lies in the inherent inhibitory effect of the product CoA on SpNatA (Gottlieb and Marmorstein, 2019). As already mentioned, CoA does not accumulate in the assay because it is immediately recycled in Ac-CoA by PDH, ensuring constant concentration of Ac-CoA. This therefore prevents the reaction from any possible inhibitory effect of CoA on the putative studied GNAT. This effect could also be associated to progressive decrease of the Ac-CoA concentration in the radioactive assay, which is not saturating and, decreases as the reaction progresses (see below). As a result, any decrease of Ac-CoA leads to a decrease of the velocity. By investigating further this phenomenon, we noticed that the concentration of Ac-CoA used in the assay was tightly associated with the quality of the raw data and signal to noise ratio. The lower the Ac-CoA concentration, the better the kinetics (Supplementary Figure 1B). The background noise—observed at high Ac-CoA concentrations—was assigned to the presence of free CoA allowing PDH to produce NADH without SpNatA. Free CoA can originate either from the Ac-CoA solution (only >93% pure at most in the stock solution) or from the hydrolysis of Ac-CoA in the reaction mixture. Indeed the Ac-CoA in stock solutions is preserved from hydrolysis by maintaining a slightly acidic pH; therefore, following mix in the neutral to slightly alkaline pH (8.0) reactor, hydrolysis of Ac-CoA may occur. As shown in Supplementary Figure 1B, a near-saturating amount of Ac-CoA of 500 μM induced a low signal-to-noise ratio as well as the establishment of the stationary phase duration giving the curves a sigmoid shape. This made uneasy the assessment of the initial velocity rate. In an attempt to optimize the assay, we used sub-saturating amounts of Ac-CoA (100 μM). Such conditions resulted in significant increase of the quality of the data and improved fit to the Michaelis–Menten equation. The newly calculated kinetic parameters for the reference peptide using 100 μM Ac-CoA in the coupled PDH assay, 9.4 ± 1.7 s^−1^ and 1.0 ± 0.3 mM (Table 1; Figure 1A) respectively, showed a *K_m_* value three times higher than that previously reported, and led to catalytic efficiency reduced by 2-fold compared to the value obtained at 500 μM Ac-CoA (Table 1). We finally observed that the catalytic efficiency determined with the coupled PDH assay at 100 μM Ac-CoA was almost identical to that assessed with the radioactive assay at 500 μM (9,279 vs. 8,800 M^−1^.s^−1^; Table 1). This indicated that SpNatA catalysis was working as efficiently in either assay, whatever the relative Ac-CoA concentration.

**Table 1:** Kinetic parameters of SpNatA with various substrates and three different assays.

Assay	[Ac-CoA] (μM)	Substrate	*k_cat_* (s^−1^)	*K_m_* (mM)	*k_cat_*/*K_m_* (M^−1^.s^−1^)
PDH	500	SASEAGVRWGRPVGRRRRP	8.9 ± 1.0	0.47 ± 0.11	19,099 ± 4,993
PDH	100	SASEAGVRWGRPVGRRRRP	9.4 ± 1.7	1.0 ± 0.3	9,279 ± 3,220
^14^C-Ac-CoA	500	SASEAGVRWGRPVGRRRRP	3.0 ± 0.5^$^	0.34 ± 0.05^$^	8,823 ± 1,638
DTNB^£^	100	SASEAGVRWGRPVGRRRRP	1.0 ± 0.1	0.36 ± 0.01	2,778 ± 288
PDH	12.5–500	SASEAGVRWGRPVGRRRRP	10.0 ± 0.4^*^	0.020 ± 0.003^*^	507,614 ± 89,931
PDH	100	SASE	>1.2	> > 10	88 ± 5
PDH	100	SASEAGV	9.8 ± 1.0	25.5 ± 3.3	384 ± 63
PDH	100	SASEAGVR	9.9 ± 1.1	14.8 ± 2.4	669 ± 131
PDH	100	SPTPAGVR	nm	nm	<3
PDH	100	SASEAGVAEQVKKLSVNDS	33.4 ± 1.5	2.3 ± 0.2	14,630 ± 1,344

$ Data from Liszczak et al. (2013).

£ Very poor initial velocity was observed (see Supplementary Figure 1C).

* Measured at saturating concentrations of the reference peptide (see Figure 1).

### Impact of Ac-CoA Concentrations on SpNatA

As the *k_cat_* values obtained with 100 and 500 μM of Ac-CoA were about the same, we wondered if the *K_m_* value of 59 μM for Ac-CoA previously determined for SpNatA was similar if measured with the PDH assay, which escaped product inhibition compared to the radioactive assay. As the peptide previously used to determine the *K_m_* for Ac-CoA was the poly-R tailed reference peptide, we used it for comparison purposes. Although the peptide could not be incubated at saturating amounts because of its inhibition effect on the PDH (see below), in the attempt to obtain the most accurate *K_m_* value for Ac-CoA, we performed several preliminary experiments for several concentration of Ac-CoA. An array of peptide concentrations was used to determine the initial velocity (*v*_i_) and fit the Michaelis–Menten equation, providing a *v*_i_/[*E*] value for each Ac-CoA concentration that reflects the value that would have been obtained with a saturating amount of peptide (Figure 1).

Of note, in kinetic analysis of multiple-substrate enzymes, the single-substrate study of Michaelis–Menten experiments is available only at saturating amounts of the second substrate; otherwise, the obtained kinetic parameters are only apparent values conditioned by both the concentration used and the *K_m_* value of the second substrate. Our data show that the *K_m_* for Ac-CoA is 20 ± 3 μM, three times lower than the value obtained with the radioactive assay. Therefore, we recommend the use of a concentration of Ac-CoA of 100–150 μM when using the PDH coupled assay with any unknown GNAT. At 100 μM, this ensures reaching >83% of the maximal theoretical velocity (*V_max_*) of SpNatA, while optimizing the quality of the kinetics and minimizing the effect of the background noise observed at the highest Ac-CoA concentrations (see previous section). Note that whatever the assay considered up to now, only apparent *V_max_* values are obtained as the Ac-CoA is not saturating; for instance, saturation is 89% with the radioactive assay at 500 μM Ac-CoA.

### DTNB Cannot Be Used for the Purpose of Screening and Discovering New GNATs

In an attempt to validate the viability of the PDH coupled assay, we challenged the results with another, already established continuous assay, relying on the reactivity of the Ellman’s reagent (5,5′-dithio-bis 2-nitrobenzoic acid, DTNB) with CoA (Kohlhaw, 1988). Reduction of the disulfide bond of DTNB to TNB allows simple continuous monitoring with a spectrophotometer at 412 nm. This assay was previously used with a number of acetyltransferases, including lysine acetyltransferases (Bode et al., 1993), p300 HAT (Thompson et al., 2004), human NAA50 (Foyn et al., 2017), *A. thaliana* and *human* NAA60 (see the section “Materials and Methods” in Støve et al. (2016) and Linster et al. (2020), and *A. thaliana* and *Candida albicans* NatB (Hong et al., 2017; Huber et al., 2020). Because of the large reactivity of DTNB, such an assay requires that the reaction mixture only contains traces of any thiol-reactive agent such as dithiothreitol or 2-mercaptoethanol and any non-thiol reducing agent cleaving disulfide bonds such as Tris(2-carboxyethyl)phosphine (TCEP). Despite these restraints, this assay is highly valuable as it is also almost independent of peptide sequence although cysteine-containing peptides need to be avoided. We could check with the PDH coupled assay that the specific activity of the enzyme assayed in the absence of reducing agents was unchanged. In contrast, when using the DTNB assay, time-course absorbance monitoring indicated that initial velocity never properly established and that the reaction rate was slowing down at least 100-fold more rapidly over time than in the case of the PDH coupled assay (Supplementary Figure 1C). This suggested rapid reaction of DTNB with an important cysteine side-chain of SpNatA, which modification induced inhibition. Pseudo-initial velocity rates could only be determined in the very first seconds of the reaction. The SpNatA catalytic efficiency as measured with the DTNB-based assay was one third of the coupled PDH and radioactive assay data (Supplementary Figure 1D; Table 1).

The high reactivity of DTNB with at least one exposed cysteine residue on the surface of SpNatA might indeed promote dissociation of the two subunits. An inhibitory effect of DTNB was previously described for other members of the GNAT family (Rudnick et al., 1992). We concluded that the DTNB assay was most risky to be used *a priori*, as it might promote biased conclusions depending on the GNAT used. DTNB was therefore useful in a case-by-case study, but only provided it has been validated with another assay before, as it was achieved with AtNAA60 (Linster et al., 2020). Therefore, DTNB coupled assay is not recommended in primary screening and discovery of any new GNAT activity. Nevertheless, a discontinuous version of the DTNB assay can be used provided the GNAT is purified and active without any reducing agent. This was successfully achieved for screening and assessing inhibitors of human NAA10 and NAA50 (Foyn et al., 2013) and assays of the *Saccharomyces cerevisiae* NatC complex (Grunwald et al., 2020).

### Influence of Peptide Length and Composition on SpNatA Activity

**Figure 2:**
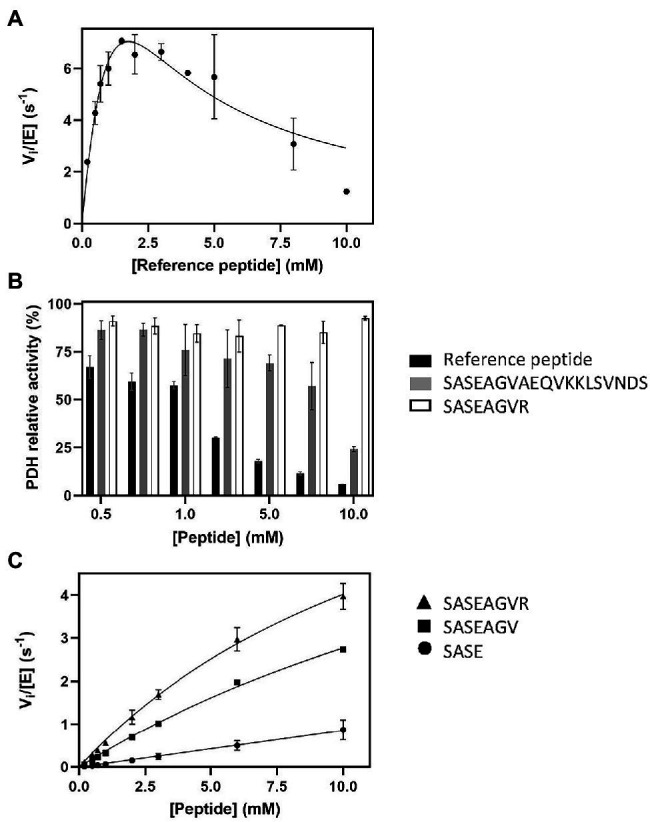
Influence of peptide sequence and composition on SpNatA and PDH activity. **(A)** Same as Figure 1A at 100 μM Ac-CoA but with higher peptide concentrations ranging from 0.1 to 10 mM. The Haldane’s equation for substrate inhibition was used to fit the data. **(B)** Inhibitory effect of various peptide substrates on PDH activity. The relative remaining PDH activity is displayed at increasing concentrations of peptides SASEAGVR (white), SASEAGVAEQVKKLSVNDS (gray), and reference peptide SASEAGVRWGRPVGRRRRP (black). **(C)** Peptides of various lengths (four, seven, and eight residues) were assayed with SpNatA in the presence of 100 μM Ac-CoA, and the data were fitted to the Michaelis–Menten equation. The peptides are SASE (Circle), SASEAGV (square), and SASEAGVR (triangle).

The above data further demonstrated the need of a continuous assay for NAT studies but also that the coupled PDH assay needed further investigation for validation. We therefore increased the peptide concentration above the previous upper value of 1 mM in an attempt to characterize more precisely the kinetic parameters. Surprisingly, higher concentrations resulted in a decrease of the initial velocity rate (Figure 2A). As the data poorly fitted with the equation for substrate inhibition model (Cornish-Bowden, 1995), this suggested another explanation to the phenomenon (Figure 2A). We, therefore, investigated the hypothesis that the reference peptide could directly interfere with PDH activity due to its polybasic C-tail tag. PDH alone was mixed in presence of increasing peptide concentrations (up to 10 mM) and its activity measured in the presence of PDH. The data showed that beyond 1 mM, the reference peptide indeed caused critical loss of PDH activity (Figure 2B).

**Figure 3:**
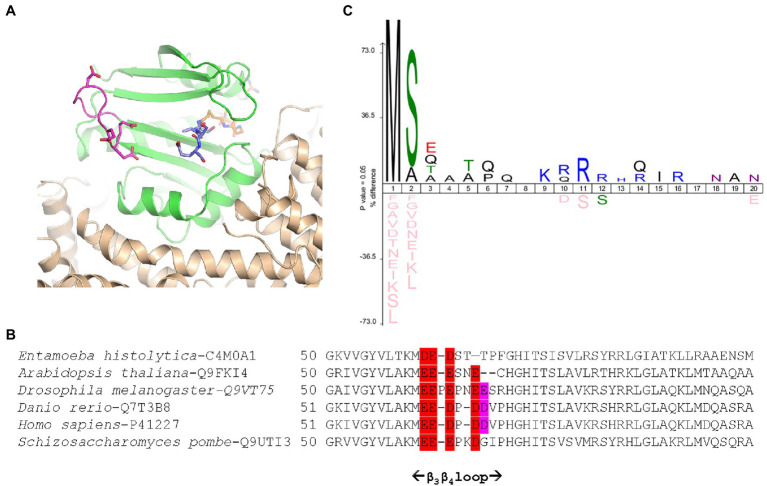
*Schizosaccharomyces pombe* NatA senses remote basic residues through a dedicated and conserved acidic stretch. **(A)** Cartoon representation of SpNatA 3D structure in complex with a bisubstrate inhibitor (PDB ID 4 kvm) showing the vicinity of an electronegative patch (displayed in panel **B**) of the NAA10 catalytic subunit with the substrate peptide moiety. SpNatA heterodimer consisting of NAA10 and NAA15 subunits is colored in green and beige, respectively. CoA and SASE peptide moieties of bisubstrate inhibitor are shown as sticks and are colored in orange and blue, respectively. The conserved residues Glu61, Glu62, Glu63, and Asp66 belonging to β_3_β_4_ loop (magenta) and forming the electronegative patch on NAA10 catalytic subunit are shown as sticks. **(B)** Sequence alignment of six NAA10 subunits aligned with Clustal Omega (Sievers et al., 2011) shows the sequence around the β_3_β_4_ loop. The extent of the loop (i.e., residues 51–69 in SpNaa10) is reported below. The electronegative patch conserved among species is colored in red with additional less conserved residues in purple. The position of the first residue of each sequence displayed is indicated. The specie and corresponding UniProt Identifier code are indicated before the sequence. **(C)** Icelogo of the first 20 residues of all natural SpNatA substrates identified by proteomics in *Saccharomyces cerevisiae* (Arnesen et al., 2009) showing the enrichment in Arg or Lys residues at positions 9–16.

The crystal structure of SpNatA in complex with a bisubstrate inhibitor (Liszczak et al., 2013) shows that the binding and selectivity of NAA10 catalytic subunit of SpNatA mostly rely on the first four amino acids of the peptide target substrate (Figure 3A). The tail tag of the reference peptide, which is used for the detection of the product in the course of the radioactive assay or for HPLC-separation purposes, is not required for the PDH coupled assay. Therefore, we performed the assay with shorter peptides devoid of the poly-R tag. Unlike the reference peptide, these peptides poorly inhibit PDH (Figure 2B) and could be used at higher concentrations in the assay. The 7- and 8-mer peptides SASEAGV and SASEAGV*R* showed much higher *K_m_* values (19 and 12 mM, respectively; Table 1; Figure 2C), while displaying identical turnover numbers (10 s^−1^). Trimming the reference peptide eventually from 19 to 7 residues led to a 10-fold reduction of the catalytic efficiency. We next used an octapeptide featuring two prolines at positions 2 and 4. Such residues are known to promote inhibition of NatA reactivity (Van Damme et al., 2011). We could verify that this peptide indeed is not a substrate of NatA, as expected (Table 1). The data indicated that octapeptides fully reconstitute NatA specificity; this result could be verified only thanks to the PDH coupled assay as the radioactive assay requires a long poly-R tail. The data also suggested that either the tail tag or the length of the peptide, or both played a critical role in increasing the binding of the enzyme to its substrates. To investigate this hypothesis, we first analyzed the acetylation of a 4-mer peptide SASE. The results showed that this peptide, though still a substrate, was less prone to catalysis than longer peptides (Table 1; Figure 2C). Thus, the catalytic efficiency of SpNatA is positively correlated with peptide size. This showed that peptide length critically affects the ability of SpNatA to acetylate any polypeptide substrate (Table 1).

Because the poly-R tail has been widely used—not only with the radioactive assay—in the NAT literature—we next investigated its effect on the catalytic efficiency of SpNatA. We tested a peptide with the same length but originating from the natural sequence of yeast protein Ths1p. Compared to the reference peptide, this natural 19-amino acid N-terminal sequence (SASEAGVAEQVKKLSVNDS) is weakly charged, eliminating the probable interference with the PDH that we assigned to the strong basic character of the tail tag. This peptide showed indeed decreased inhibitory effect on PDH compared the reference peptide (Figure 2B). The data showed the catalytic efficiency was 1.5 fold higher, with the associated *k_cat_* of the neutral peptide increased by a factor of 3.5 with respect to the reference, basic peptide. In contrast, the *K_m_* value was two-fold higher than that of the reference peptide (Table 1). This suggested that the basic character of the C-tail improved peptide affinity to SpNatA. A structural analysis of the protein SpNatA revealed that a strongly negatively charge labile β_3_β_4_ loop, located at the extremity of the peptide binding site, nicely explains this increased affinity, as the Arg residues could form salt bridges with the conserved four aspartates and glutamates of the loop (Figures 3A,B). It is also interesting that yeast substrates of NatA show enriched composition starting with Ser and Ala to a smaller extent, followed by small chain amino acids, and feature one or two basic residues at positions ranging between 9 and 16 (Figure 3C). Taken together, these results show that the first four N-terminal residues, which determine enzyme selectivity, are not sufficient to promote both sufficient substrate binding and the highest efficiency to SpNatA. This impact was so far unknown as it was missed from early analysis only focused on the first five residues of the acetylated sequence (see Figure 1C in Colaert et al., 2009). This long-range effect could be unveiled and experimentally confirmed thanks to the new PDH coupled assay.

### Identification of a New GNAT (OsGNAT2) From Rice

In order to check the validity of the PDH coupled assay and apply its relevance to the discovery of novel GNATs, we chose a homolog of the *A. thaliana* GNAT within the NAA90 family, the latest NAA family described so far (Giglione and Meinnel, 2021). This homolog from rice (*O. sativa*) was pointed out recently and surmised to display similar activity as AtGNAT2 (Table 2 in Giglione and Meinnel, 2021). This GNAT remains yet uncharacterized. It shows the highest homology with AtGNAT2 and we referred to it as OsGNAT2. Like AtGNAT2, OsGNAT exhibits an N-terminal extension predicted to act as a plastid-targeting signal. We cloned OsGNAT2 and expressed the protein devoid of its N-terminal targeting pre-sequence. OsGNAT2 was purified to homogeneity in frame with MBP fused at the C-side. The purified protein was used in the PDH coupled assay along with 100 μM Ac-CoA, the SpNatA reference peptide, and a significant activity could be detected (Table 2). OsGNAT2 specific activity was very similar to that of AtGNAT2 obtained with a different assay and with a dissimilar peptide substrate (Table 2). In contrast, very low or no activity was observed with natural amino acids (Lys and Arg) or metabolites (serotonin, 5-methoxytryptamine), which are classic small substrates of several GNATs (Table 2). This indicated that OsGNAT2 works on polypeptide and behaves as a NAT. Upon release of the His-MBP tag following TEV cleavage, we observed that OsGNAT2 devoid of MBP displayed an activity very similar to that of the MBP fused protein on the Tsh1p unmodified peptide (35 vs. 24 nmol.min^−1^.μmol^−1^, respectively). This indicated that the MBP tag did not influence the activity of the fused GNAT. Next, we observed that the Tsh1p unmodified peptide was far less efficient than the reference peptide with both forms. This indicated that OsGNAT2 likely displays specificity determinants in the far-remote C-terminal side as SpNatA does. Finally, we observed no significant improvement of the catalytic efficiency when increasing Ac-CoA concentration up to 500 μM, indicative of a *K_m_* value of Ac-CoA lower than 30 μM. We concluded that the 100 μM Ac-CoA concentration selected for SpNatA was also perfectly suited for OsGNAT2.

**Table 2:** OsGNAT2 specific activity is similar to AtGNAT2 and displays unique features.

Peptide substrate	Peptide length	Specific NTA activity (nmol.min^−1^.μmol^−1^)^$^
SASEAGVRWGRPVGRRRRP	19	19 ± 1
TAQGA[Ac-K]AA[Dnp-K]-Ahx-r-r-r-NH_2_^£^	8^£^	32^*^
SASEAGV	7	0.2 ± 0.1
SASEAGVAEQVKKLSVNDS	19	0.5 ± 0.1
Lysine	1	0.1 ± 0.1
Arginine	1	0.6 ± 0.1
Serotonin	1	<0.02
5-Methoxytryptamine	1	0.4 ± 0.1
TQTF	4	21 ± 0.5
TQTFIP	6	19 ± 0.4
TQTFIPGK	8	33 ± 0.9
TQTFIPGKDA	10	24 ± 0.9
TQTFIPGKDAALEDS	15	19 ± 0.6
TQTFIPGKDAALEDSIARFQQK	22	30 ± 1
TQTFIPGSDAALEDSIASFQQS	22	13 ± 1
TQTFIPGKDARWGRPVGRRRRP	22	757 ± 42
AVAANKR	7	1.4 ± 0.3
AVAANKRSVM	10	11 ± 0.3
SNSYDSS	7	5.2 ± 0.3
SNSYDSSSIK	10	9.9 ± 0.3
MNMPMTERIR	10	22 ± 1
RTNPTTS	7	<0.02
RTNPTTSNPE	10	<0.02

$ All values were measured with the PDH-coupled assay in the presence of 100 μM Ac-CoA and of His-MBP-GNAT2 fusion (1–20 μM).

£ Dnp is for di-nitro phenyl substitution on the ε-amino group, Ahx is an aminohexanoic spacer, and *r* is for D-Arg (see Seidel et al., 2016).

* Data from Bienvenut et al. (2020), measured with Maltose Binding Protein (MBP)-AtGNAT2 fusion.

### OsGNAT2 Displays Unusual Substrate Specificity

**Figure 4:**
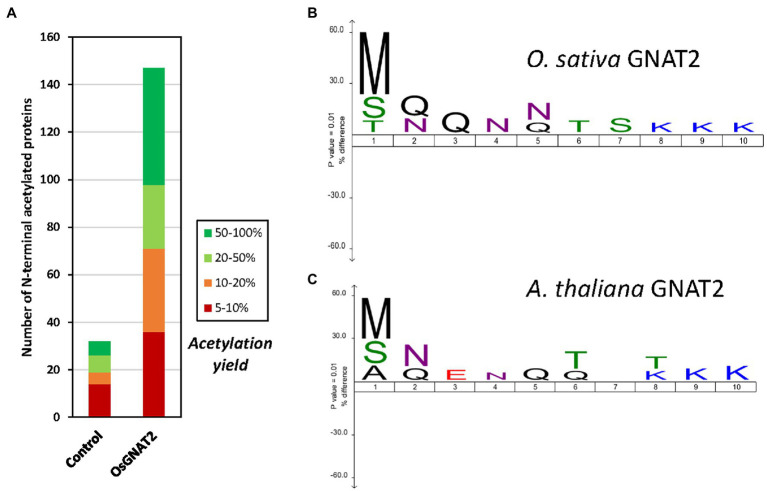
OsGNAT2 is active as a N-acetyltransferase (NAT) and displays unusual sequence features. **(A)** Increase of the number of acetylated N-termini assessed by Global Analysis Profiling (GAP) assay following induced overexpression OsGNAT2 in *Escherichia coli* by comparison to a control overexpressing AtNaa15. Colors show the yield of acetylated N-termini. **(B)** Icelogo (Colaert et al., 2009) showing the composition of the first 10 residues of the identified acetylated N-termini from the GAP assay with >20% acetylation compared to the composition of the *E. coli* proteome showing the enrichment in Arg and Lys residues at positions 8–10. The size of the letter in the logo is proportional. **(C)** Same as **(B)** but with the AtGNAT2 dataset (Bienvenut et al., 2020).

To further challenge the N-terminal activity of OsGNAT2, we performed a GAP assay. This assay consists in expression of the NAT-MBP fusion in *E. coli*, an organism where few, and well-known proteins undergo NTA and which basic level is known (Bienvenut et al., 2015; Dinh et al., 2015; Schmidt et al., 2016). Next, the SILProNAQ pipeline, dedicated to proteome range quantitative analysis of protein N-termini, was applied (Table 3). The complete list of 1,299 non-redundant protein N-terminal peptides revealed by the GAP assay is compiled in Supplementary Dataset 1. Among them, 753 were quantified (368 in the control and 385 when OsGNAT2 was expressed). The data reveal an increase not only of the number of NTAed substrates but also of the average NTA yield of the N-termini when cells were grown in the presence of OsGNAT2 (Table 3; Figure 4A). Forty-nine *E. coli* proteins showed a huge increase of their NTA yield resulting from the expression of OsGNAT2. Figure 4B shows that OsGNAT2, like AtGNATs, exhibits a relaxed substrate specificity similar to Archaeal NATs (Liszczak and Marmorstein, 2013; Bienvenut et al., 2020) and that the first N-terminal amino acid is the major determinant (M, S, or T are retrieved but also A, I, and V; Table 3). Like SpNatA the following residues appear to be uncharged and hydrophilic. We cannot formally exclude that the observed relaxed specificity results from heterologous production of OsGNAT2 involving incomplete or heterologous folding of the recombinant protein (Baneyx and Mujacic, 2004; González-Montalbán et al., 2007; Rosano and Ceccarelli, 2014). Nevertheless, this relaxed specificity of both OsGNAT2 and AtGNAT2—was similar to that deduced *in planta* from the N-acetylation defects detected in the *gnat2* knockout *A. thaliana* line (Bienvenut et al., 2020). This further confirmed the accuracy of the GAP analysis for plastid GNAT substrate specificity analysis, as already observed with the cytosolic plant forms (see Giglione and Meinnel, 2021 and references therein). The GAP assay allows to determine 21 proteins strongly acetylated by both OsGNAT2 (Supplementary Dataset 1) and AtGNAT2 (Bienvenut et al., 2020). OsGNAT2 and AtGNAT2 display therefore tightly overlapping specificity determinants featuring little preference for the N-terminal residue (Figures 4B,C). It is therefore most likely that both fulfill similar physiological role.

**Table 3:** Main proteins substrates of OsGNAT as assessed from the GAP assay.

Uniprot ID	Entry name	Protein description	NTA position	Preceding residue	N-acetylated sequence^a^	%Ac in control^b^	%Ac with OsGNAT2^b^
P0ABS1	DKSA	RNA polymerase-binding transcription factor DksA	1	-	MQEGQNRKTS	0.2	100.0
P00579	RPOD	RNA polymerase sigma factor RpoD (Sigma-70)	1	-	MEQNPQSQLK	0.8 ± 0.3	99.8 ± 0.1
P0AES6	GYRB	DNA gyrase subunit B (EC 5.6.2.2)	2	M	SNSYDSSSIK	2.4 ± 0.7	98.7 ± 0.2
P08244	PYRF	Orotidine 5′-phosphate decarboxylase (EC 4.1.1.23)	2	M	TLTASSSSRA	0.1 ± 0.0	96.1 ± 1.5
P30850	RNB	Exoribonuclease 2 (EC 3.1.13.1)	1	-	MFQDNPLLAQ	3.5 ± 0.5	98.8 ± 1.4
P0A8Z0	YCIA	Acyl-CoA thioester hydrolase YciA (EC 3.1.2.-; Protein P14)	2	M	STTHNVPQGD	0.3 ± 0.0	92.5 ± 2.7
P67603	AC4CH	N(4)-acetylcytidine amidohydrolase (EC 3.5.1.135)	1	-	MQPNDITFFQ	3.3 ± 4.1	91.1 ± 2.8
P0A6Z1	HSCA	Chaperone protein HscA (Hsc66)	2	M	ALLQISEPGL	8.0 ± 0.5	95.7 ± 0.9
P40191	PDXK	Pyridoxine/pyridoxal/pyridoxamine kinase (EC 2.7.1.35)	2	M	SSLLLFNDKS	12.2 ± 0.7	99.8 ± 0.1
P52067	FSR	Fosmidomycin resistance protein	2	M	AMSEQPQPVA	9.8 ± 1.1	97.3 ± 1.9
P0ACB0	DNAB	Replicative DNA helicase (EC 3.6.4.12)	2	M	AGNKPFNKQQ	0.6 ± 0.3	88.1 ± 5.3
P0ACD4	ISCU	Iron–sulfur cluster assembly scaffold protein IscU	2	M	AYSEKVIDHY	0.5 ± 0.2	87.0 ± 0.7
P0A734	MINE	Cell division topological specificity factor	2	M	ALLDFFLSRK	0.5 ± 0.3	86.6
P00452	RIR1	Ribonucleoside-diphosphate reductase 1 subunit alpha (EC 1.17.4.1)	1	-	MNQNLLVTKR	0.2 ± 0.1	83.4 ± 4.7
P0ABB0	ATPA	ATP synthase subunit alpha (EC 7.1.2.2)	1	-	MQLNSTEISE	0.5 ± 0.3	83.6 ± 9.5
P0AAE0	CYCA	D-serine/D-alanine/glycine transporter	2	M	VDQVKVVADD	39.8	82.7 ± 1.6
P75838	YCAO	Ribosomal protein S12 methylthiotransferase accessory factor YcaO	2	M	TQTFIPGKDA	1.0 ± 0.6	81.4 ± 1.2
P0A9S3	GATD	Galactitol 1-phosphate 5-dehydrogenase (EC 1.1.1.251)	1	-	MKSVVNDTDG	0.3 ± 0.1	78.9 ± 11.3
P0ACA3	SSPA	Stringent starvation protein A	2	M	AVAANKRSVM	0.2 ± 0.0	77.8 ± 7.2
P0AF08	APBC	Iron–sulfur cluster carrier protein	1	-	MNEQSQAKSP	0.8 ± 0.0	73.8 ± 4.3
P77549	YFCJ	Uncharacterized MFS-type transporter YfcJ	2	M	TAVSQTETRS	5.5 ± 0.2	73.8 ± 7.0
P60716	LIPA	Lipoyl synthase (EC 2.8.1.8)	2	M	SKPIVMERGV	0.5 ± 0.2	70.4
P0A8V2	RPOB	DNA-directed RNA polymerase subunit beta (EC 2.7.7.6)	2	M	VYSYTEKKRI	1.4 ± 0.7	64.8 ± 2.3
P27434	RODZ	Cytoskeleton protein RodZ	1	-	MNTEATHDQN	0.4 ± 0.1	63.0 ± 6.8
P69222	IF1	Translation initiation factor IF-1	2	M	AKEDNIEMQG	1.0 ± 0.1	63.4 ± 1.7
P0A7X3	RS9	30S ribosomal protein S9	1	-	MAENQYYGTG	0.2 ± 0.0	52.5 ± 6.4
P60438	RL3	50S ribosomal protein L3	2	M	IGLVGKKVGM	0.6 ± 0.1	52.5 ± 9.7
P22255	CYSQ	3′(2′),5′-bisphosphate nucleotidase CysQ (EC 3.1.3.7)	1	-	MLDQVCQLAR	1.2	52.4
P0AGJ5	YFIF	Uncharacterized tRNA/rRNA methyltransferase YfiF (EC 2.1.1.-)	1	-	MNDEMKGKSG	1.3	50.8 ± 0.1
P0ABT2	DPS	DNA protection during starvation protein (EC 1.16.-.-)	2	M	STAKLVKSKA	2.8	47.4
P07012	RF2	Peptide chain release factor RF2 (RF-2)	1	-	MFEINPVNNR	0.7 ± 0.1	44.4 ± 6.4

a Protein N-termini selected for *in vitro* analysis are colored in gray.

b MBP-GNAT fusions were expressed in *Escherichia coli* and protein N-terminal Ac yields determined using the SILProNAQ approach. Protein entries displaying significant NTA increase (>40%) after induction of OsGNAT2 in *E. coli* compared to control samples expressing AtNaa15 non catalytic subunit of AtNatA were selected and displayed; when the SD is missing this means the entry was quantified only once among the three replicates.

We selected decapeptide substrate representatives from the *E. coli* most strongly acetylated proteins in the OsGNAT2 GAP assay (grayed in Table 3) and exhibiting various classes of N-termini, namely A, S, and T. Among them, TQTFIPGKDA derived from the N-terminus of ribosomal protein S12 methylthiotransferase accessory factor (YCAO, P75838). SNSYDSSSIK derived from DNA gyrase subunit B (GYRB, P0AES6). AVAANKRSVM derived from stringent starvation protein (SSPA; P0ACA3). The three peptides were tested with the PDH coupled assay. Although we observed a high *K_m_* value preventing to reach the *V_max_*, the data indicated NTA activities of OsGNAT2 for the three peptides were comparable to that of the reference peptide (Table 2). However, these small peptides showed much improved performances compared to those of the reference NatA peptide devoid of the poly-R tail (SASEAGV and SASEAGVAEQVKKLSVNDS; Table 2). To additionally validate our GAP test, we characterized N-terminal peptides derived from a protein, which NTA was not affected by the expression of OsGNAT2 in bacteria (RTNPTTS and RTNPTTSNPE). As expected, the assayed peptides, independently of their length, were not substrates of OsGNAT2 in our PDH assay (Table 2).

We next observed that peptide length appeared to have a relatively poor effect on OsGNAT2 catalysis (Table 2). About 4- to 22-residues long peptides of the YCAO series indeed displayed similar activity (Table 2). The substrate specificity of OsGNAT2 appears therefore to be fully contained into the very first residues. This is unlike SpNatA, which needs long peptides to gain full efficiency. We therefore searched for other longer-range impacts. As the OsGNAT2 substrates displayed a similar pattern as the ones for SpNatA, with positively charged residues at positions 9–10, we challenged the influence of the basic character of the peptide on the catalytic parameters. To do so, we tested the 22 amino acid-long peptide TQTFIPGKDARWGRPVGRRRRP, featuring the basic C-tag fused to the TQTFIPGKDA YCAO substrate. The measured activity was 25 times higher than that of the 22-residues long peptide derived from the natural sequence (Table 2). This indicated that OsGNAT2 was very sensitive to the occurrence of basic residues remote from the N-terminus. This was finally confirmed by changing the three remote positive charges of YCAO 22-mer and the associated reduced catalytic rate associated with the new peptide variant (Table 2).

Overall, our data clearly indicate that, like SpNatA, efficient catalysis with OsGNAT2 requires both well suited N-terminal sequence and most likely also remote positively charged residues. Furthermore, this suggests that a negatively-charged patch functionally corresponding to the β_2_β_4_ loop of SpNatA (Figure 3) could be involved in the binding of substrates with a positively charged region. One such patch does occur at the N-terminus of OsGNAT2 between positions 83 and 95 (APIEEEEPLPEE). The role of remote positive charges as supplemental pattern contributing to substrate selection of GNAT2 in addition to the key role of very N-terminal residues is supported by the sequence logo, which was retrieved from the GAP study performed with either *A. thaliana* or *O. sativa* GNAT2. Indeed, polylysine tracks are clearly identified between positions 8 and 10 of both patterns (Figures 4B,C).

To finally check whether our data were fully in line with the logo displayed in Figure 4B, we selected a peptide sequence which could not be quantified in our analysis but which sequence did match the pattern shown in Figure 4B. We selected the MNMPMTERIR decapeptide. This peptide displays no internal K residue and therefore should bring unambiguous answer through the PDH-coupled assay. The sequence was derived from galactoside O-acetyltransferase (THGA, P07464). We could verify that the derived N-terminal decapeptide was indeed a substrate of OsGNAT2 with catalytic efficiency similar to the other decapeptides (Table 2).

**Figure 5:**
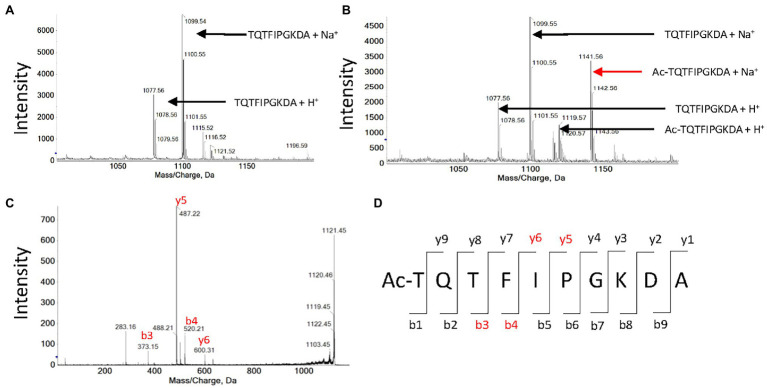
MALDI-Tof analysis of peptide TQTFIPGKDA acetylation by OsGNAT2. **(A)** Mass spectrum obtained after an hour incubation of 1 mM TQTFIPGKDA peptide in the absence of OsGNAT2. **(B)** Same as panel **(A)** in the presence of 10 μM OsGNAT2. **(C)** MS/MS spectrum obtained after an hour incubation of 1 mM peptide TQTFIPGKDA in the presence of 10 μM OsGNAT2. MS/MS analysis of the 1,141 Da peak in panel **(B)**. **(D)** Expected proteotypic ions following the fragmentation in MS/MS of Ac-TQTFIPGKDA peptide (ProteinProspector, https://prospector.ucsf.edu). Peaks found on the spectra C are colored in red.

We reported that plastid GNATs like OsGNAT2 display dual NAT and KAT activity. In the previous paragraph, several peptides devoid of K residues were substrates of OsGNAT2, in favor of NAT activity of the GNAT. Nevertheless, a number of selected peptides feature a K in their sequence. For instance, the YCAO decapeptide displays both a free N-terminus and an internal lysine at position 8. Therefore, we attempted to discriminate the N-acetylation type(s) OsGNAT2 ensured by mass spectrometry. After incubation of the peptide in the presence of OsGNAT2, MALDI-Tof mass spectrometry analysis revealed that the peptide was acetylated only in the presence of Ac-CoA and OsGNAT2 (Figures 5A,B). Next, fragmentation of the acetylated product of the reaction by MS/MS revealed the presence of four proteotypic ions of the N-terminal acetylated peptide only (Figures 5C,D). We could not observe any proteotypic ion of the K acetylation even after long incubation at higher concentrations of OsGNAT2. This does not mean that OsGNAT2 does not display KAT activity but rather that KAT substrates need yet to be discovered. We cannot exclude that fully folded proteins could be more appropriate substrates than peptides for this purpose. Such substrates would exhibit the reactive Lysine in a better-suited environment, which mimic the post-translational KAT activity of GNATs.

## Conclusion

In this report, we describe a novel robust coupled assay for GNAT assessment. The assay was validated with the very challenging SpNatA heterodimer and with a new type of GNAT of the NAA90 family, OsGNAT2, featuring relaxed specificity. We show that the assay is easy, can be performed on a simple bench spectrometer but is also high throughput scalable as it can be used with 96-well plate. Reaction can be started with a pipetting robot distributing Ac-CoA. This automated assay can be alternatively used in the future not only for determining NTA status of any GNAT candidate but also for assessing NTA inhibitors. Overall, this new assay is a new tool aimed at much better understanding of the number and function of GNATs, substrates, and inhibitors.

## Data Availability Statement

Mass spectrometry proteomics data associated to the GAP assay have been uploaded to the ProteomeXchange Consortium^2^ via the PRIDE repository^3^ (Vizcaíno et al., 2013) with the dataset identifier PXD030189.

## Author Contributions

TA conducted all kinetic analyses. CD and TA performed purification of NatA and OsGNAT2. J-BB performed GAP analysis. FR performed MALDI mass spectrometry experiments. TM, CD, and CG conceived the project and supervised the experiments. TM, CD, CG, and TA analyzed the data. CG and TM wrote the manuscript with contributions of TA and CD. All authors contributed to the article and approved the submitted version.

## Funding

This work was supported by KatNat (ERA-NET, ANR-17-CAPS-0001-01) and CanMore (France-Germany PRCI, ANR-20CE92-0040) grants funded by French National Research Agency (ANR), and Fondation ARC (ARCPJA320200600002137). This work has benefited from the support of a French State grant (ANR-17-EUR-0007, EUR SPS-GSR) managed by the ANR under an Investments for the Future program (ANR-11-IDEX-0003-02), and from the facilities and expertise of the I2BC proteomic platform SICaPS, supported by IBiSA, Ile de France Region, Plan Cancer, CNRS and Paris-Sud University. TA is supported by a PhD studentship from the Paris-Saclay University. FR is supported by grants from Région Ile-de-France (17012695) and Foundation pour la Recherche Médicale (FDT202001010779).

## Conflict of Interest

The authors declare that the research was conducted in the absence of any commercial or financial relationships that could be construed as a potential conflict of interest.

## Publisher’s Note

All claims expressed in this article are solely those of the authors and do not necessarily represent those of their affiliated organizations, or those of the publisher, the editors and the reviewers. Any product that may be evaluated in this article, or claim that may be made by its manufacturer, is not guaranteed or endorsed by the publisher.

## Footnotes

^1^

^2^

^3^

## References

[ref1] Aebersold R. Mann M. (2016). Mass-spectrometric exploration of proteome structure and function. Nature 537, 347–355. doi: 10.1038/nature19949, PMID: 27629641

[ref2] Aksnes H. Drazic A. Marie M. Arnesen T. (2016). First things first: vital protein marks by N-terminal acetyltransferases. Trends Biochem. Sci. 41, 746–760. doi: 10.1016/j.tibs.2016.07.005, PMID: 27498224

[ref3] Aksnes H. Ree R. Arnesen T. (2019). Co-translational, post-translational, and non-catalytic roles of N-terminal acetyltransferases. Mol. Cell 73, 1097–1114. doi: 10.1016/j.molcel.2019.02.007, PMID: 30878283 PMC6962057

[ref4] Appelmans O. Kashyap R. S. Gilles P. De Borggraeve W. M. Voet A. Van Lint J. (2021). LSA-50 paper: An alternative to P81 phosphocellulose paper for radiometric protein kinase assays. Anal. Biochem. 630:114313. doi: 10.1016/j.ab.2021.114313, PMID: 34302798

[ref5] Arnesen T. Van Damme P. Polevoda B. Helsens K. Evjenth R. Colaert N.. (2009). Proteomics analyses reveal the evolutionary conservation and divergence of N-terminal acetyltransferases from yeast and humans. Proc. Natl. Acad. Sci. U. S. A. 106, 8157–8162. doi: 10.1073/pnas.0901931106, PMID: 19420222 PMC2688859

[ref6] Baneyx F. Mujacic M. (2004). Recombinant protein folding and misfolding in *Escherichia coli*. Nat. Biotechnol. 22, 1399–1408. doi: 10.1038/nbt1029, PMID: 15529165

[ref7] Berndsen C. E. Denu J. M. (2005). Assays for mechanistic investigations of protein/histone acetyltransferases. Methods 36, 321–331. doi: 10.1016/j.ymeth.2005.03.002, PMID: 16085424

[ref8] Berndsen C. E. Denu J. M. (2008). Catalysis and substrate selection by histone/protein lysine acetyltransferases. Curr. Opin. Struct. Biol. 18, 682–689. doi: 10.1016/j.sbi.2008.11.004, PMID: 19056256 PMC2723715

[ref9] Bienvenut W. V. Brünje A. Boyer J.-B. Mühlenbeck J. S. Bernal G. Lassowskat I.. (2020). Dual lysine and N-terminal acetyltransferases reveal the complexity underpinning protein acetylation. Mol. Syst. Biol. 16:e9464. doi: 10.15252/msb.20209464, PMID: 32633465 PMC7339202

[ref10] Bienvenut W. V. Giglione C. Meinnel T. (2015). Proteome-wide analysis of the amino terminal status of *Escherichia coli* proteins at the steady-state and upon deformylation inhibition. Proteomics 15, 2503–2518. doi: 10.1002/pmic.201500027, PMID: 26017780

[ref11] Bienvenut W. V. Giglione C. Meinnel T. (2017a). SILProNAQ: a convenient approach for proteome-wide analysis of protein N-termini and N-terminal acetylation quantitation. Methods Mol. Biol. 1574, 17–34. doi: 10.1007/978-1-4939-6850-3_3, PMID: 28315241

[ref12] Bienvenut W. V. Scarpelli J. P. Dumestier J. Meinnel T. Giglione C. (2017b). EnCOUNTer: a parsing tool to uncover the mature N-terminus of organelle-targeted proteins in complex samples. BMC Bioinformatics 18:182. doi: 10.1186/s12859-017-1595-y, PMID: 28320318 PMC5359831

[ref13] Bode R. Thurau A. M. Schmidt H. (1993). Characterization of acetyl-CoA: L-lysine N6-acetyltransferase, which catalyses the first step of carbon catabolism from lysine in *Saccharomyces cerevisiae*. Arch. Microbiol. 160, 397–400. doi: 10.1007/bf00252227, PMID: 8257283

[ref14] Boisson B. Meinnel T. (2003). A continuous assay of myristoyl-CoA:protein N-myristoyltransferase for proteomic analysis. Anal. Biochem. 322, 116–123. doi: 10.1016/j.ab.2003.07.007, PMID: 14705787

[ref15] Colaert N. Helsens K. Martens L. Vandekerckhove J. Gevaert K. (2009). Improved visualization of protein consensus sequences by iceLogo. Nat. Methods 6, 786–787. doi: 10.1038/nmeth1109-786, PMID: 19876014

[ref16] Cornish-Bowden A. (1995). “Inhibition and activation of enzymes,” in Fundamentals of Enzyme Kinetics (London, UK: Portland Press Ltd.), 93–128.

[ref17] Deng S. Marmorstein R. (2021). Protein N-terminal acetylation: structural basis, mechanism, versatility, and regulation. Trends Biochem. Sci. 46, 15–27. doi: 10.1016/j.tibs.2020.08.005, PMID: 32912665 PMC7749037

[ref18] Dian C. Pérez-Dorado I. Rivière F. Asensio T. Legrand P. Ritzefeld M.. (2020). High-resolution snapshots of human N-myristoyltransferase in action illuminate a mechanism promoting N-terminal Lys and Gly myristoylation. Nat. Commun. 11:1132. doi: 10.1038/s41467-020-14847-3, PMID: 32111831 PMC7048800

[ref19] Dinh T. V. Bienvenut W. V. Linster E. Feldman-Salit A. Jung V. A. Meinnel T.. (2015). Molecular identification and functional characterization of the first Nalpha-acetyltransferase in plastids by global acetylome profiling. Proteomics 15, 2426–2435. doi: 10.1002/pmic.201500025, PMID: 25951519 PMC4692087

[ref20] Drazic A. Myklebust L. M. Ree R. Arnesen T. (2016). The world of protein acetylation. Biochim. Biophys. Acta 1864, 1372–1401. doi: 10.1016/j.bbapap.2016.06.007, PMID: 27296530

[ref21] Dyda F. Klein D. C. Hickman A. B. (2000). GCN5-related N-acetyltransferases: a structural overview. Annu. Rev. Biophys. Biomol. Struct. 29, 81–103. doi: 10.1146/annurev.biophys.29.1.81, PMID: 10940244 PMC4782277

[ref22] Evjenth R. Hole K. Ziegler M. Lillehaug J. R. (2009). Application of reverse-phase HPLC to quantify oligopeptide acetylation eliminates interference from unspecific acetyl CoA hydrolysis. BMC Proc. 3:S5. doi: 10.1186/1753-6561-3-s6-s5, PMID: 19660098 PMC2722098

[ref23] Evjenth R. H. Van Damme P. Gevaert K. Arnesen T. (2013). HPLC-based quantification of in vitro N-terminal acetylation. Methods Mol. Biol. 981, 95–102. doi: 10.1007/978-1-62703-305-3_7, PMID: 23381855

[ref24] Foyn H. Jones J. E. Lewallen D. Narawane R. Varhaug J. E. Thompson P. R.. (2013). Design, synthesis, and kinetic characterization of protein N-terminal acetyltransferase inhibitors. ACS Chem. Biol. 8, 1121–1127. doi: 10.1021/cb400136s, PMID: 23557624

[ref25] Foyn H. Thompson P. R. Arnesen T. (2017). DTNB-based quantification of in vitro enzymatic N-terminal acetyltransferase activity. Methods Mol. Biol. 1574, 9–15. doi: 10.1007/978-1-4939-6850-3_2, PMID: 28315240

[ref26] Giglione C. Meinnel T. (2021). Evolution-driven versatility of N terminal acetylation in photoautotrophs. Trends Plant Sci. 26, 375–391. doi: 10.1016/j.tplants.2020.11.012, PMID: 33384262

[ref27] González-Montalbán N. García-Fruitós E. Villaverde A. (2007). Recombinant protein solubility—does more mean better? Nat. Biotechnol. 25, 718–720. doi: 10.1038/nbt0707-718 17621288

[ref28] Gottlieb L. Marmorstein R. (2019). Biochemical and structural analysis of N-terminal acetyltransferases. Methods Enzymol. 626, 271–299. doi: 10.1016/bs.mie.2019.07.016, PMID: 31606079 PMC6884420

[ref29] Grunwald S. Hopf L. V. M. Bock-Bierbaum T. Lally C. C. M. Spahn C. M. T. Daumke O. (2020). Divergent architecture of the heterotrimeric NatC complex explains N-terminal acetylation of cognate substrates. Nat. Commun. 11:5506. doi: 10.1038/s41467-020-19321-8, PMID: 33139728 PMC7608589

[ref30] Hamel L. D. Deschenes R. J. Mitchell D. A. (2014). A fluorescence-based assay to monitor autopalmitoylation of zDHHC proteins applicable to high-throughput screening. Anal. Biochem. 460, 1–8. doi: 10.1016/j.ab.2014.05.013, PMID: 24878334 PMC6445550

[ref31] Ho Y. H. Chen L. Huang R. (2021). Development of a continuous fuorescence-based assay for N-terminal acetyltransferase D. Int. J. Mol. Sci. 22:594. doi: 10.3390/ijms22020594, PMID: 33435607 PMC7827481

[ref32] Hong H. Cai Y. Zhang S. Ding H. Wang H. Han A. (2017). Molecular basis of substrate specific acetylation by N-terminal acetyltransferase NatB. Structure 25, 641–649.e3. doi: 10.1016/j.str.2017.03.003, PMID: 28380339

[ref33] Huber M. Bienvenut W. V. Linster E. Stephan I. Armbruster L. Sticht C.. (2020). NatB-mediated N-terminal acetylation affects growth and abiotic stress responses. Plant Physiol. 182, 792–806. doi: 10.1104/pp.19.00792, PMID: 31744933 PMC6997699

[ref34] Kang K. Lee K. Park S. Byeon Y. Back K. (2013). Molecular cloning of rice serotonin N-acetyltransferase, the penultimate gene in plant melatonin biosynthesis. J. Pineal Res. 55, 7–13. doi: 10.1111/jpi.12011, PMID: 22998587

[ref35] Khoury G. A. Baliban R. C. Floudas C. A. (2011). Proteome-wide post-translational modification statistics: frequency analysis and curation of the swiss-prot database. Sci. Rep. 1:90. doi: 10.1038/srep00090, PMID: 22034591 PMC3201773

[ref36] Kim Y. Tanner K. G. Denu J. M. (2000). A continuous, nonradioactive assay for histone acetyltransferases. Anal. Biochem. 280, 308–314. doi: 10.1006/abio.2000.4546, PMID: 10790315

[ref37] Kohlhaw G. B. (1988). Alpha-isopropylmalate synthase from yeast. Methods Enzymol. 166, 414–423. doi: 10.1016/s0076-6879(88)66054-x 3071716

[ref38] Krtenic B. Drazic A. Arnesen T. Reuter N. (2020). Classification and phylogeny for the annotation of novel eukaryotic GNAT acetyltransferases. PLoS Comput. Biol. 16:e1007988. doi: 10.1371/journal.pcbi.1007988, PMID: 33362253 PMC7790372

[ref39] Linster E. Layer D. Bienvenut W. V. Dinh T. V. Weyer F. A. Leemhuis W.. (2020). The *Arabidopsis* Nα-acetyltransferase NAA60 locates to the plasma membrane and is vital for the high salt stress response. New Phytol. 228, 554–569. doi: 10.1111/nph.16747, PMID: 32548857

[ref40] Linster E. Stephan I. Bienvenut W. V. Maple-Grodem J. Myklebust L. M. Huber M.. (2015). Downregulation of N-terminal acetylation triggers ABA-mediated drought responses in Arabidopsis. Nat. Commun. 6:7640. doi: 10.1038/ncomms8640, PMID: 26184543 PMC4530475

[ref41] Liszczak G. Goldberg J. M. Foyn H. Petersson E. J. Arnesen T. Marmorstein R. (2013). Molecular basis for N-terminal acetylation by the heterodimeric NatA complex. Nat. Struct. Mol. Biol. 20, 1098–1105. doi: 10.1038/nsmb.2636, PMID: 23912279 PMC3766382

[ref42] Liszczak G. Marmorstein R. (2013). Implications for the evolution of eukaryotic amino-terminal acetyltransferase (NAT) enzymes from the structure of an archaeal ortholog. Proc. Natl. Acad. Sci. U. S. A. 110, 14652–14657. doi: 10.1073/pnas.1310365110, PMID: 23959863 PMC3767502

[ref43] Magin R. S. Liszczak G. P. Marmorstein R. (2015). The molecular basis for histone H4- and H2A-specific amino-terminal acetylation by NatD. Structure 23, 332–341. doi: 10.1016/j.str.2014.10.025, PMID: 25619998 PMC4318724

[ref44] Polevoda B. Sherman F. (2003). N-terminal acetyltransferases and sequence requirements for N-terminal acetylation of eukaryotic proteins. J. Mol. Biol. 325, 595–622. doi: 10.1016/s0022-2836(02)01269-x, PMID: 12507466

[ref45] Rathore O. S. Faustino A. Prudencio P. Van Damme P. Cox C. J. Martinho R. G. (2016). Absence of N-terminal acetyltransferase diversification during evolution of eukaryotic organisms. Sci. Rep. 6:21304. doi: 10.1038/srep21304, PMID: 26861501 PMC4748286

[ref46] Rosano G. L. Ceccarelli E. A. (2014). Recombinant protein expression in *Escherichia coli*: advances and challenges. Front. Microbiol. 5:172. doi: 10.3389/fmicb.2014.00172, PMID: 24860555 PMC4029002

[ref47] Rudnick D. A. Duronio R. J. Gordon J. I. (1992). “Methods for studying myristoyl-coenzyme A: protein N-myristoyltransferase,” in Lipid Modification by Proteins. A Practical Approach. eds. Hooper N. M. Turner A. J. (Oxford: IRL Press), 37–61.

[ref48] Salah Ud-Din A. I. Tikhomirova A. Roujeinikova A. (2016). Structure and functional diversity of GCN5-related N-acetyltransferases (GNAT). Int. J. Mol. Sci. 17:E1018. doi: 10.3390/ijms17071018, PMID: 27367672 PMC4964394

[ref49] Schmidt A. Kochanowski K. Vedelaar S. Ahrné E. Volkmer B. Callipo L.. (2016). The quantitative and condition-dependent *Escherichia coli* proteome. Nat. Biotechnol. 34, 104–110. doi: 10.1038/nbt.3418, PMID: 26641532 PMC4888949

[ref50] Seidel J. Klockenbusch C. Schwarzer D. (2016). Investigating deformylase and deacylase activity of mammalian and bacterial sirtuins. Chembiochem 17, 398–402. doi: 10.1002/cbic.201500611, PMID: 26708127

[ref51] Sievers F. Wilm A. Dineen D. Gibson T. J. Karplus K. Li W.. (2011). Fast, scalable generation of high-quality protein multiple sequence alignments using clustal omega. Mol. Syst. Biol. 7:539. doi: 10.1038/msb.2011.75, PMID: 21988835 PMC3261699

[ref52] Silverstein R. M. (1975). The determination of the molar extinction coefficient of reduced DTNB. Anal. Biochem. 63, 281–282. doi: 10.1016/0003-2697(75)90219-5, PMID: 1111072

[ref53] Støve S. I. Magin R. S. Foyn H. Haug B. E. Marmorstein R. Arnesen T. (2016). Crystal structure of the Golgi-associated human Nα-acetyltransferase 60 reveals the molecular determinants for substrate-specific acetylation. Structure 24, 1044–1056. doi: 10.1016/j.str.2016.04.020, PMID: 27320834 PMC4938767

[ref54] Thompson P. R. Wang D. Wang L. Fulco M. Pediconi N. Zhang D.. (2004). Regulation of the p300 HAT domain via a novel activation loop. Nat. Struct. Mol. Biol. 11, 308–315. doi: 10.1038/nsmb740, PMID: 15004546

[ref55] To T. L. Piggott B. J. Makhijani K. Yu D. Jan Y. N. Shu X. (2015). Rationally designed fluorogenic protease reporter visualizes spatiotemporal dynamics of apoptosis in vivo. Proc. Natl. Acad. Sci. U. S. A. 112, 3338–3343. doi: 10.1073/pnas.1502857112, PMID: 25733847 PMC4371907

[ref56] Traverso J. A. Giglione C. Meinnel T. (2013). High-throughput profiling of N-myristoylation substrate specificity across species including pathogens. Proteomics 13, 25–36. doi: 10.1002/pmic.201200375, PMID: 23165749

[ref57] Van Damme P. Evjenth R. Foyn H. Demeyer K. De Bock P. J. Lillehaug J. R.. (2011). Proteome-derived peptide libraries allow detailed analysis of the substrate specificities of N(alpha)-acetyltransferases and point to hNaa10p as the post-translational actin N(alpha)-acetyltransferase. Mol. Cell. Proteomics 10:004580. doi: 10.1074/mcp.M110.004580, PMID: 21383206 PMC3098586

[ref58] Vetting M. W. de Carvalho S. L. P. Yu M. Hegde S. S. Magnet S. Roderick S. L.. (2005). Structure and functions of the GNAT superfamily of acetyltransferases. Arch. Biochem. Biophys. 433, 212–226. doi: 10.1016/j.abb.2004.09.003, PMID: 15581578

[ref59] Vizcaíno J. A. Côté R. G. Csordas A. Dianes J. A. Fabregat A. Foster J. M.. (2013). The PRoteomics IDEntifications (PRIDE) database and associated tools: status in 2013. Nucleic Acids Res. 41, D1063–D1069. doi: 10.1093/nar/gks1262, PMID: 23203882 PMC3531176

[ref60] Westrich L. D. Gotsmann V. L. Herkt C. Ries F. Kazek T. Trösch R.. (2020). The versatile interactome of chloroplast ribosomes revealed by affinity purification mass spectrometry. Nucleic Acids Res. 49, 400–415. doi: 10.1093/nar/gkaa1192, PMID: 33330923 PMC7797057

