# Supplementary material for: A Continuous Assay Set to Screen and Characterize Novel Protein N-Acetyltransferases Unveils Rice General Control Non-repressible 5-Related N-Acetyltransferase2 Activity
Source: Front Plant Sci. 2022 Feb 22;13:832144. doi: 10.3389/fpls.2022.832144 (PMC8902505; doi:10.3389/fpls.2022.832144)
Supplement: Supplementary file 1 [file Presentation_1.pdf]

Article collection on "Plant Protein Termini : Their Generation, Modification and Function »

A continuous assay to characterize novel protein N-acetyltransferases unveils rice GNAT2 activity

Thomas Asensio[1], Cyril Dian[1,iD], Jean-Baptiste Boyer[1,id], Frédéric Rivière[1,iD], Thierry Meinnel[1,iD,*] and Carmela Giglione[1,iD,*]

[1] *Université Paris Saclay, CEA, CNRS, Institute for Integrative Biology of the Cell (I2BC), 91198 Gif-sur-Yvette cedex, France*

[iD] ORCID : 0000-0002-1702-3300 (T. Asensio); 0002-6349-3901 (C. Dian); 0000-0001-5265-3917 (J.-B. Boyer); 0000-0001-6366-1904 (F. Rivière); 0001-5642-8637 (T. Meinnel); 0002-7475-1558 (C. Giglione).

*** Correspondence:** Carmela Giglione, carmela.giglione@i2bc.paris-saclay.fr or Thierry Meinnel, thierry.meinnel@i2bc.paris-saclay.fr

SUPPLEMENTARY MATERIAL

Supplementary Figure 1. Superior performances and reliability of the PDH- with respect to DTNB- assay.

(**A**) Initial velocity rates of the acetylation reaction measured at varying SpNatA concentration. Concentration of both AcCoA and reference peptide concentrations were set at 0.5 mM and the assay was performed presence of 150 $mU.mL^{-1}$ of PDH. Linearity between enzyme concentration and observed velocity rate as shown with the straight line is no longer followed above 0.2 $\mu M^{-1}.s^{-1}$. (**B**) NADH release followed by absorbance at 340 nm resulting from SpNatA activity in the presence of the reference peptide substrate at two different concentrations of AcCoA 100 µM (triangles) and 500 µM AcCoA (circles) in absence (open dots) or presence (filled dots) of 1 mM reference peptide. (**C**) Product formation followed by absorbance at 412 nm following DTNB reaction with CoA (circles) or 340 following PDH reaction forming NADH (triangles) after SpNatA catalysis in presence of 1 mM reference peptide. The trend curve for the DTNB reaction was fitted with the seven first data points of the reaction (120 seconds) and all the data for the first 1000 seconds for the PDH coupled reaction. The scale for DTNB (right) is ten-fold lower than for PDH (left). (**D**) SpNatA displays reduced activity towards reference peptide SASEAGVRWGRPVGRRRRP when assessed with the DTNB assay. Michaelis-Menten equation is used to fit the data of DTNB assay (circles) and PDH assay (triangles).

Supplementary Figure 2. Long-range effects of the sequence peptide on OsGNAT activity

OsGNAT2 kinetics with different substrate peptides fit to the Michaelis-Menten equation. Plots were the average of three independent experiments data sets performed in the presence of 100 µM AcCoA with varying concentrations of peptides TQTFIPGKDA (black circles) and TQTFIPGKDARWGRPVGRRRRP (black squares). NB: Significant inhibition of PDH activity was observed with the longest peptide at concentrations over 1 mM.
