# Supplementary figures and images for: A Continuous Assay Set to Screen and Characterize Novel Protein N-Acetyltransferases Unveils Rice General Control Non-repressible 5-Related N-Acetyltransferase2 Activity
Source: Front Plant Sci. 2022 Feb 22;13:832144. doi: 10.3389/fpls.2022.832144 (PMC8902505; doi:10.3389/fpls.2022.832144)

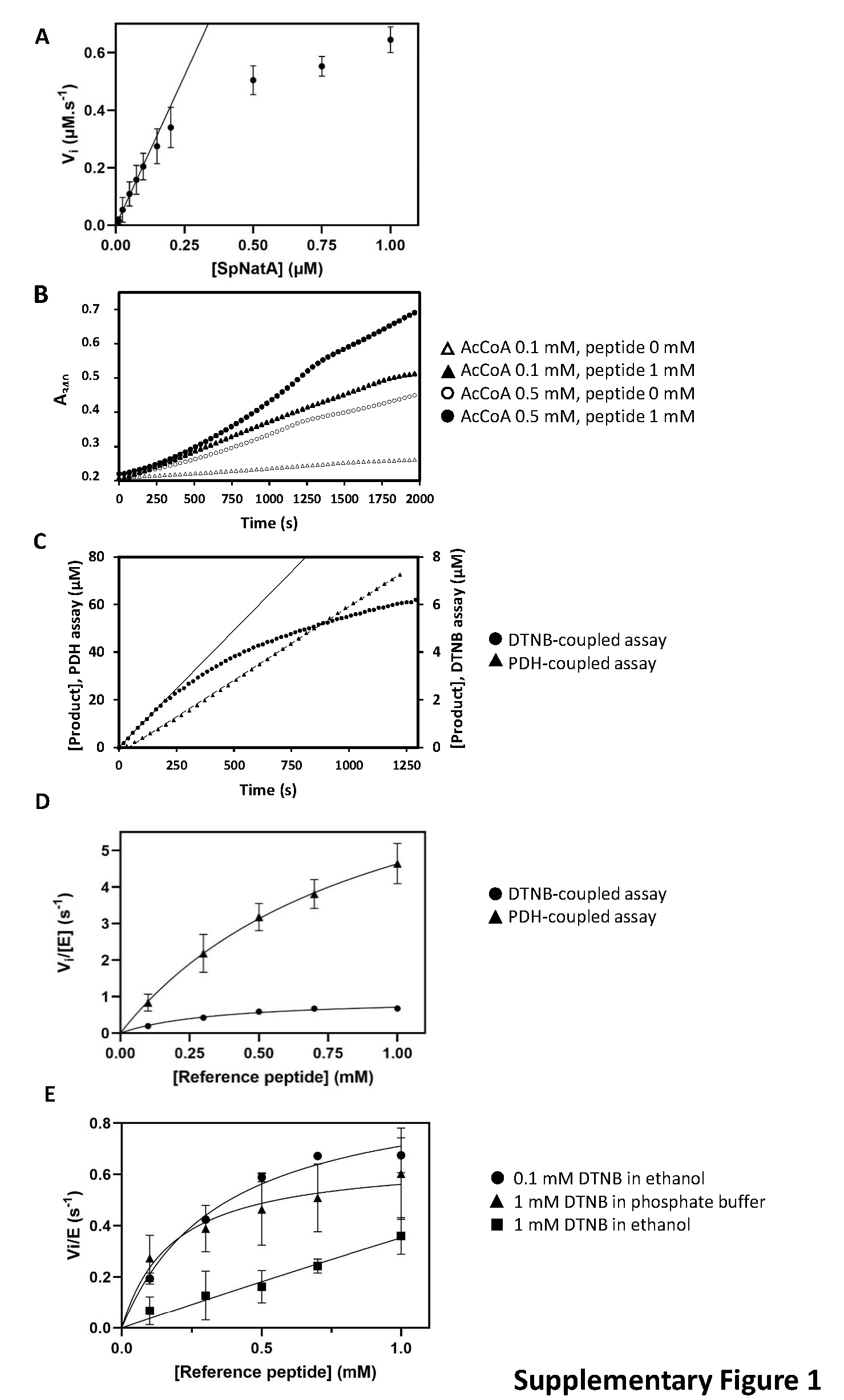

Supplement: Supplementary file 2 [file Image_1.tif]

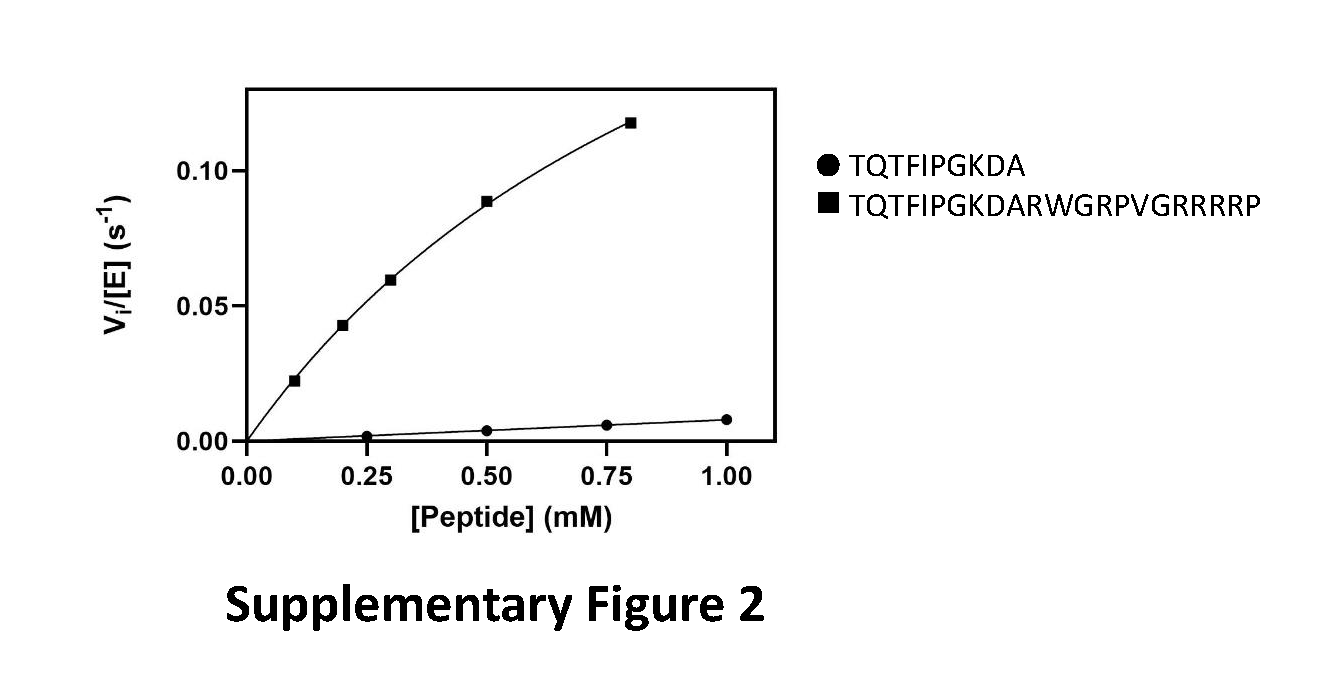

Supplement: Supplementary file 3 [file Image_2.tif]
